# Study on the construction technology of β-alanine synthesizing *Escherichia coli* based on cellulosome assembly

**DOI:** 10.3389/fbioe.2023.1202483

**Published:** 2023-06-02

**Authors:** Jie Lu, Guodong Wang, Cuiping Yang, Zehao Peng, Lu Yang, Bowen Du, Chuanzhuang Guo, Songsen Sui, Jianbin Wang, Junlin Li, Ruiming Wang, Junqing Wang

**Affiliations:** ^1^ State Key Laboratory of Biobased Material and Green Papermaking (LBMP), Qilu University of Technology, Jinan, Shandong, China; ^2^ Department of Biological Engineering, Qilu University of Technology, Jinan, Shandong, China; ^3^ Zhucheng Dongxiao Biotechnology Co., Ltd., Zhucheng, Shandong, China

**Keywords:** β-alanine, gene knockout, cellulosome, dockerin, cohesin, enzymatic catalysis

## Abstract

**Introduction:** β-Alanine is the only β-amino acid in nature; it is widely used in food additives, medicines, health products, and surfactants. To avoid pollution caused by traditional production methods, the synthesis of β-alanine has been gradually replaced by microbial fermentation and enzyme catalysis, which is a green, mild, and high-yield biosynthesis method.

**Methods:** In this study, we constructed an *Escherichia coli* recombinant strain for efficient β-alanine production using glucose as the raw material. The microbial synthesis pathway of L-lysine-producing strain, *Escherichia coli* CGMCC 1.366, was modified using gene editing by knocking out the aspartate kinase gene, *lysC*. The catalytic efficiency and product synthesis efficiency were improved by assembling key enzymes with cellulosome.

**Results:** By-product accumulation was reduced by blocking the L-lysine production pathway, thereby increasing the yield of β-alanine. In addition, catalytic efficiency was improved by the two-enzyme method to further increase the β-alanine content. The key cellulosome elements, dockerin (*docA*) and cohesin (*cohA*), were combined with L-aspartate-α-decarboxylase (*bspanD*) from *Bacillus subtilis* and aspartate aminotransferase (*aspC*) from *E.coli* to improve the catalytic efficiency and expression level of the enzyme. β-alanine production reached 7.439 mg/L and 25.87 mg/L in the two engineered strains. The β-alanine content reached 755.465 mg/L in a 5 L fermenter.

**Discussion:** The content of β-alanine synthesized by constructed β-alanine engineering strains were 10.47 times and 36.42 times higher than the engineered strain without assembled cellulosomes, respectively. This research lays the foundation for the enzymatic production of β-alanine using a cellulosome multi-enzyme self-assembly system.

## 1 Introduction

β-Alanine is a neutral amino acid that constitutes the structure of a human protein. It is widely used in the food, medicine, and chemical industries (Blancquaert et al., 2017; Pandurangan et al., 2017). It can be used as a food additive, sports nutrition supplement, and feed to improve production performance (Belity et al., 2022; Murphy et al., 2022; Wang et al., 2022). It can be prepared by chemical synthesis and biotechnology, which currently includes whole-cell biocatalysis, enzyme catalysis, and metabolic engineering with microbial fermentation (Wang et al., 2021a). Enzyme-catalyzed methods have received increasing attention compared to other methods in recent decades. β-Alanine is an important amino acid derived from L-aspartic acid. In the enzymatic synthesis of β-alanine, L-aspartic acid-α-decarboxylase (Zhao et al., 2022) was used for catalytic synthesis of β-alanine through decarboxylation of L-aspartate. The method is environmentally friendly, inexpensive and has fewer by-products. Aspartate decarboxylases have been extensively studied, and are mainly derived from *Escherichia coli*, *Corynebacterium glutamicum*, *Bacillus subtilis*, and *Mycobacterium tuberculosis* (Yu et al., 2020). Whole cells co-expressing L-aspartate-α-decarboxylase from *B. subtilis* and L-aspartate-α-decarboxylase from *Tribolium castaneum* catalyzed fumaric acid to generate β-alanine (Wang L. et al., 2020). Multi-enzyme co-expression improves transformation efficiency; however, the raw material cost is relatively high. L-aspartate-α-decarboxylase of *B. subtilis* was introduced into *E. coli* to construct a β-alanine-producing strain, and the inactivation of the L-aspartate-α-decarboxylase mechanism was eliminated by changing the central carbon metabolic flux and other technologies, adopting the fermentation process for production (Li et al., 2022a). The easy inactivation of L-aspartate-α-decarboxylase is overcome by mutation.

**FIGURE 1:**
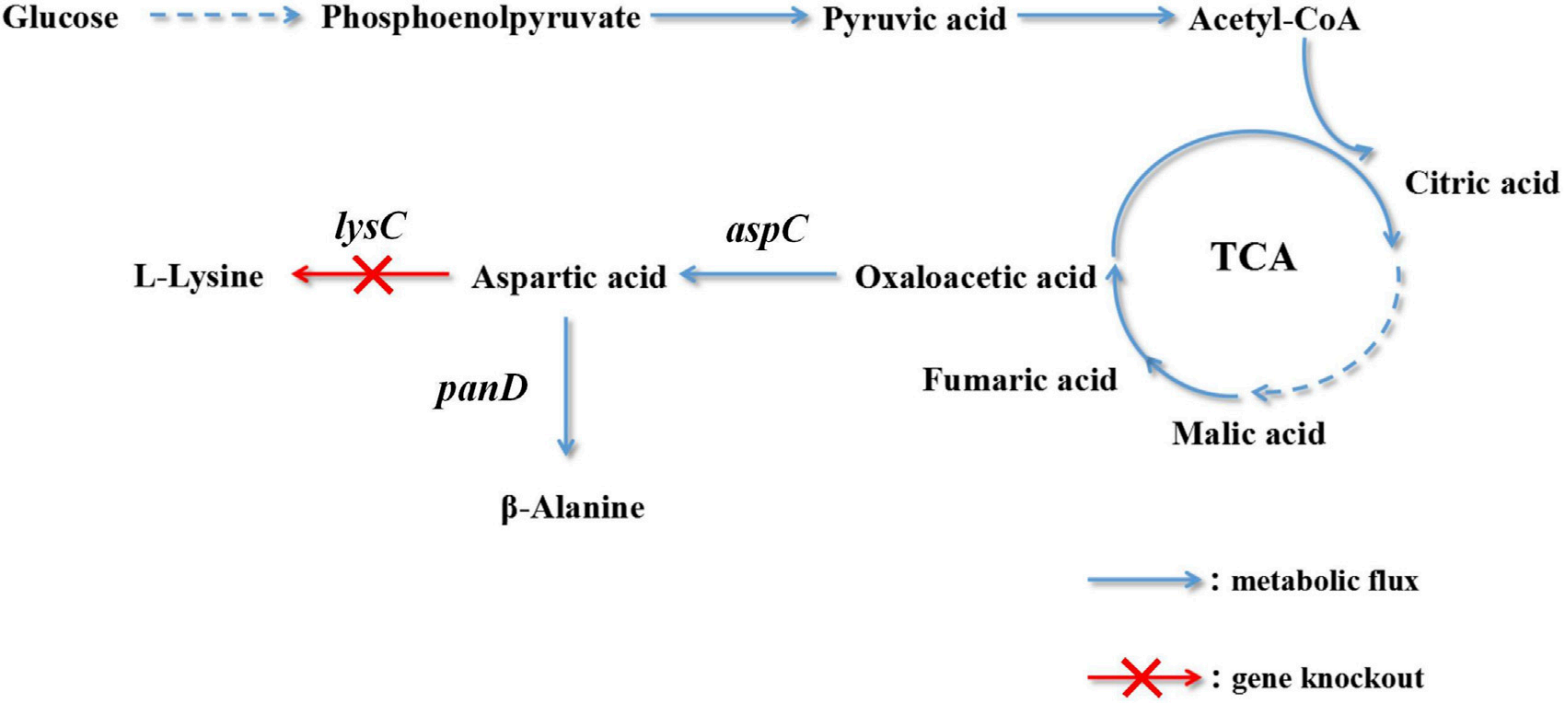
Pathway engineering for β-alanine production in recombinant *Escherichia coli*.

The metabolic synthesis of β-alanine is promoted by knocking out bypass genes. The aspartic acid content is increased by overexpressing aspartate dehydrogenase genes to further improve the yield of β-alanine. Oxaloacetate biosynthesis is enhanced by high expression of the aspartate dehydrogenase gene and the introduction of pyruvate decarboxylase (Zou et al., 2020). The biosynthetic pathway of *C. glutamicum* engineered bacteria to produce L-lysine was modified. The optimal enzyme for improving the yield of β-alanine was selected: L-aspartate-α-decarboxylase of *B. subtilis.* Lactate dehydrogenase and alanine/valine transaminase were destroyed, and by-product accumulation was reduced. L-Aspartate-α-decarboxylase was mutated to increase its expression (Wang et al., 2021b). There are few reports on the production of β-alanine by the fermentation of *C. glutamicum*. This production technology provides new ideas and a foundation. In this study, the metabolic pathway of *E. coli* was modified to generate a yield of L-lysine. The biosynthesis of L-lysine and β-alanine has the same precursor, L-aspartic acid. L-Lysine accumulation was reduced using gene knockout technology, and L-aspartic acid was directed to the biosynthesis pathway of β-alanine (Figure 1).

Cellulosomes are natural multi-enzyme self-assembly systems that can organize and coordinate multiple enzyme components to efficiently catalyze and degrade lignocellulose. They are mainly comprised of two parts: dockerin (*docA*) and cohesin (*cohA*). The functional domain of dockerin can connect one end of the enzyme involved in assembly and interact with cohesin to form complementary modules tightly bound together (Galera-Prat et al., 2020). Cohesins contain structural proteins known as scaffold proteins (Sca). Scaffold proteins can be classified into anchoring and primary scaffold proteins. The anchoring scaffold proteins directly anchor the cellulosome to the cell surface (Hu and Zhu, 2019), and the primary scaffold proteins contain a docking protein that can combine the carbohydrate-binding module (CBM) with other scaffold proteins. The attachment of cellulosomes to cellulose substrates is determined by the carbohydrate-binding module (CBM), which shortens the distance between the substrate and enzyme component (Levi Hevroni et al., 2020). Cellulosomes have small molecular weights, many binding sites, rich docking protein types, and strong operability. This may shorten the transfer and processing of intermediate metabolites between catalytic active centers of different enzymes and allow different enzyme proteins to be assembled according to a certain proportion by means of scaffold proteins to flexibly regulate the types and expression levels of enzyme proteins and improve the catalytic efficiency of enzymes; this improves the synthesis efficiency of products (Jiang et al., 2021). Increasing number of studies in various fields have taken advantage of the benefits of cellulosomes; for example, the use of β-glucosidase coupled cellulosome complexes improved the saccharification activity on crystalline cellulose (Hirano et al., 2019). The efficiency of β-galactosidase expression on the surface of *B. subtilis* spores can be improved by the interaction between adhesion and anchor proteins (Wang et al., 2021c). A synthetic scaffold protein was constructed for lactate secretion in conjunction with the major scaffold protein of *Clostridium cellulovorans* and the domain of endoglucanase E (Tarraran et al., 2021). Efficient direct conversion of carboxymethyl cellulose to bioethanol was achieved by *in vitro* assembly of three recombinant cellulases and cell surface CBMs to construct cellulosomes assembled on the surface of *Pichia pastoris* (Dong et al., 2020). In this study, we analyzed the synthesis of L-aspartate-α-decarboxylase (*bspanD*) from *B. subtilis* and aspartate aminotransferase (*aspC)* from *E. coli* in the β-alanine biosynthesis pathway using key elements of cellulosomes: a multi-enzyme self-assembly system of dockerin (*docA*) and cohesin (*cohA*). This improves the catalytic efficiency and expression level of enzymes and further promotes β-alanine production.

## 2 Materials and methods

### 2.1 Strains, plasmids, primers, and culture conditions

An *E. coli* gene-knockout vector was constructed using pET28a (+) vector (Sangon, Shanghai, China) and cultured in Luria Broth (LB) medium at 37°C. *E. coli* DH5α (Vazyme Biotech, Nanjing, China) cells were used as hosts for gene cloning in Luria Broth (LB) medium at 37°C. The L-lysine-producing strain *E. coli* CGMCC 1.366 strain (from our laboratory) was used as the host to carry out gene cloning and culture in fermentation medium for β-alanine production. The fermentation formula for producing β-alanine by *E. coli* is as follows: 15.1 g Na_2_HPO_4_ 12H_2_O, 3 g KH_2_PO_4_, 1 g NH_4_CI, 0.5 g NaCl, 13.21 g (NH_4_)_2_SO_4_, 20 g glucose, 10.0 g FeSO_4_·7H_2_O, 1.0 g CuSO_4_·5H_2_O, 0.5 g MnSO_4_·4H_2_O, 2.3 g ZnSO_4_·7H_2_O, 0.1 g (NH_4_)Mo_7_O_24_, 0.2 g, Na_2_B_4_O_7_·10H_2_O, 2.0 g CaCl_2_ and 25 mg, L-lysine. The pH was adjusted to 7 with 100 g L^−1^ NaHCO_3_. The primers were synthesized by Sangon Biotech (Shanghai, China). All the chemicals were purchased from Sigma-Aldrich (St. Louis, MO, United States). The enzyme and plasmid preparation kits for restriction enzymes were provided by Vazyme (Nanjing, China). All the strains and plasmids used in this study are listed in Table 1. All the primers used in this study are listed in Supplementary Table S1.

**TABLE 1:** Strains and plasmids used in this study.

	Description	Source
Strains		
CGMCC 1.366	*E. coli* engineering bacteria for high-yield L-lysine	This laboratory
CGMCC 1.366-A	*E. coli* CGMCC 1.366∆*lysC*	This study
CGMCC 1.366-B	*E. coli* CGMCC 1.366∆*lysC*/pET28a (+)-*aspC*-*cohA*-*panD*-*docA*	This study
CGMCC 1.366-C	*E. coli* CGMCC 1.366∆*lysC*/pET28a (+)-*panD*-*cohA*-*aspC*-*docA*	This study
CGMCC 1.366-D	*E. coli* CGMCC 1.366∆*lysC*/pET28a (+)-*aspC*-*panD*-*docA*	This study

Plasmids		
pET28a (+)	Kan^R^, T7 promoter, f1 origin	Sangon
pKD13	Kan^R^, Ap^R^, template for amplifying FRT-kan-FRT	Sangon
pKD46	Ap^R^, λ Red recombinase expression plasmid, ara-inducible, temperature sensitive	Sangon
pCP20	Ap^R^, Cm^R^, Flp recombinase expression plasmid, temperature sensitive	Sangon
pET28a (+)-*aspC*-*cohA*-*panD*-*docA*	Kan^R^, assemble in the order of aspartate aminotransferase (*aspC*)、 cohesin (*cohA*)、L-aspartate-α-decarboxylase (*panD*) and dockerin (*docA*)	Sangon
pET28a (+)-*panD*-*cohA*-*aspC*-*docA*	Kan^R^, Kan^R^, assemble in the order of L-aspartate-α-decarboxylase (*panD*)、 cohesin (*cohA*)、 aspartate aminotransferase (*aspC*) and dockerin (*docA*)	Sangon
pET28a (+)-*aspC*-*panD*-*docA*	Kan^R^, containing aspartate aminotransferase (*aspC*), L-aspartate-α-decarboxylase (*panD*), dockerin (*docA*)	This study

### 2.2 Knockout fragment fusion technique for the *lysC* gene knockout in engineered *Escherichia coli*

The genome of *E. coli* was used as the template to search for the gene encoding aspartate kinase *lysC* (WP_203077836.1) homologous protein on the NCBI official website to determine its gene length and sequence. The aspartokinase gene (Dookeran and Nielsen, 2021) in the L-lysine-producing *E. coli* pathway was knocked out using the RED homologous recombination experiment (Chao and Hu, 2022). The upper and lower homology arms (*lysC*1 and *lysC*2) were amplified by PCR using the upper and lower homology arm primers, *lysC*1-F/R and *lysC*2-F/R, respectively, designed using the CE design software. The FRT-Kan-FRT sequence in plasmid pKD13 was amplified by PCR using the upstream and downstream primers, FRT-Kan-FRT-F/R, of the FRT site fragment with overlapping homologous arms. The amplification conditions of the three fragments were as follows: pre-denaturation at 95°C for 3 min; denaturation at 95°C for 15 s, annealing at 60°C for 15 s, extension at 72°C for 1 min using 30 cycles, and a final extension at 72°C for 5 min. Purification and recovery was performed using a gel recovery kit (Vazyme Biotech, Nanjing, China) and gene fragments were obtained, including a 500 bp fragment of *lysC*1, a 500 bp fragment of *lysC*2, and a 1,324 bp fragment of FRT-Kan-FRT. The plasmid pET28a (+) was double-digested with restriction enzymes *Nde* I and *Hin*d III (Thermo Fisher Scientific, United States), purified, and recovered (Valente et al., 2021). A one-step cloning kit was used for seamless cloning (Vazyme Biotech, Nanjing, China). The concentrations of the target genes *lysC*1, *lysC*2, and FRT-Kan-FRT recovered from the gel and expression vector pET28a (+) recovered after double enzyme digestion purification were detected using a MD2000 microspectrophotometer (UVITECe, United Kingdom). The gene fragments were diluted so that the concentration of the three target genes was 10 ng/μL, and the concentration of linearized pET28a (+) vector was 100 ng/μL. The PCR was performed at 37°C for 30 min, and the fragments obtained by overlapping polymerase chain reaction were cloned into plasmid DH5α. Individual colonies growing on the transformed plate (50 μg/mL kanamycin) were cultured in LB liquid medium with corresponding resistance markers at 37°C and 200 r/min overnight on a shaker. The pET28a (+)-*lysC*1-FRT-Kan-FRT-*lysC*2 plasmid was extracted and used as a template to amplify the knockout frame FRT-CKan-FRT with Ck-F/R upstream and downstream primers. The length of the knockout box (FRT-CKan-FRT fragment) was 2,324 bp.

### 2.3 Knockout fragment transformation technique for *lysC* gene knockout in engineered *Escherichia coli*

*E. coli* CGMCC 1.366 competent cells (Yang et al., 2022) were prepared in advance. Single colonies were inoculated in 50 mL liquid LB, and the cell density in the bacterial solution was controlled at OD_600_ = 0.2 by shaking culture at 37°C. The temperature-sensitive plasmid pKD46 containing arabinose (Liao et al., 2018) induced recombinase was introduced into the strain, and *E. coli* CGMCC 1.366 competent cells (prepared in a frozen state and stored at −80°C) were quickly taken out and placed on ice (0°C) for thawing (approximately 10–15 min). Ten microliters of pKD46 plasmid was gently added to competent cells to avoid the generation of bubbles. The bottom of the centrifuge tube was gently tapped with a finger for mixing and the tube was placed on ice for half an hour. The sample was heat shocked in a 42°C water bath for 90 s, followed by cooling on ice at 0°C for 2–3 min. Then, 900 μL of LB liquid medium was added to the centrifuge tube to recover the transformed cells. The pKD46 plasmid is temperature-sensitive; therefore, the recovery culture condition was 30°C at 200 rpm for 1 h. The bacterial solution was centrifuged at 5,000 r/min for 5 min, transferred to a sterile super-clean bench, followed by the removal of 900 μL supernatant, and the remaining liquid was used to gently resuspend the bacteria. The resuspended bacterial solution was used to spread the cells onto a LB solid medium plate (with 100 μg/mL ampicillin). The cells were incubated at 30°C, placed upright for approximately 10 min, then inverted for overnight culture. Single clones were selected as templates for colony PCR verification, and a gene fragment on the pKD46 plasmid was amplified using YZpkd46-F/R as upstream and downstream primers. A single colony that was correctly verified was cultured in LB liquid medium (with 100 μg/mL ampicillin) at 30°C and 200 r/min overnight. CGMCC 1.366/pKD46 competent cells were prepared by converting the knockout frame CK into CGMCC 1.366/pKD46 competent cells to obtain the recombinant strains CGMCC 1.366/pKD46-CK. Different single colonies growing on the above plate were selected, placed in 50 mL of corresponding resistant LB liquid medium, cultured at 30°C and 200 r/min, and knockout box Ck-F/R were used as the primers for PCR amplification verification. The verified strains (according to agarose gel electrophoresis) were selected and sent for DNA sequencing. Correctly verified clones were directly used for the next experiment. The temperature-sensitive plasmid pCP20 was transformed into the strain CGMCC 1.366/pKD46-CK to produce FLP recombinase (Xiao et al., 2022), which removed the kanamycin resistance gene. Finally, positive clones were screened by colony PCR. The homology of the fusion fragment was confirmed by DNA sequencing, and recombinant *E. coli* CGMCC 1.366-A was obtained.

### 2.4 Construction and transformation of recombinant plasmids

*panD* and *aspC* were fused to cohesin and dockerin domains to drive their sequential co-immobilization on self-assembly systems. The inducible promoter Ptrc, *aspC*, *cohA*, *panD* and *docA* were cloned into pET28a (+) plasmid by selecting restriction enzyme sites *Eco*R I and *Not* I. DocA is an intracellular mutant that has undergone activity verification. Li et al. (2022b) have confirmed that DocA can bind and assemble within cells, and improve product synthesis. The target fragment was cloned into a pET28a (+) plasmid. Two plasmids were constructed based on the different assembly modes of the key elements of the cellulosome. Plasmids pET28a (+)-*aspC*-*cohA*-*panD*-*docA* and pET28a (+)-*panD*-*cohA*-*aspC*-*docA* were synthesized by Shanghai Sangon Biotech. The unassembled recombinant plasmid pET28a (+)-*aspC*-*panD*-*docA* was constructed by inverse PCR to verify the assembly effect of cellulosomes using the recombinant plasmid pET28a (+)-*aspC*-*cohA*-*panD*-*docA* as a template, the target gene pET28a (+)-*aspC*-*panD*-*docA* was amplified by reverse PCR. The PCR amplification conditions involved predenaturation at 95°C for 3 min, denaturation at 95°C for 15 s, annealing at 60°C for 15 s, and extension at 72°C for 1 min, the process of denaturation, annealing and extension was cycled 30 times, followed by a final extension at 72°C for 5 min. The products obtained by reverse PCR amplification were digested with *Dpn* I, and the target bands were recovered using a DNA gel recovery kit (Vazyme, Nanjing, China) to obtain the recombinant plasmid pET28a (+)-*aspC*-*panD*-*docA*. The construction design of these three plasmids is shown in Supplementary Figure S1. Finally, recombinant plasmids were electrotransformed into *E. coli* CGMCC 1.366-A competent cells to construct engineered *E. coli* CGMCC 1.366-B with vector pET28a (+)-*aspC*-*cohA*-*panD*-*docA*, *E. coli* CGMCC 1.366-C with vector pET28a (+)-*panD*-*cohA*-*aspC*-*docA*, and *E. coli* CGMCC 1.366-D with vector pET28a (+)-*aspC*-*panD*-*docA*. Primers YZcohA-F/R and YZdohA-F/R were used for PCR amplification. Verification strip as shown in Supplementary Figure S2. Compare and observe the size and shape of the band presented on the DNA gel map through the band of the DNA marker used to judge whether the band is correct. The strains verified to be correct were directly subjected to the next experiment of 50% glycerol preservation.

Verification of induced expression of cohesin and dockerin: *E. coli* CGMCC 1.366-B, *E. coli* CGMCC 1.366-C, and *E. coli* CGMCC 1.366-D were inoculated in 50 mL liquid LB medium containing 50 μg/mL kanamycin and cultured at 37°C and 200 r/min in a constant temperature shaker until an OD_600_ of 0.8. Isopropyl-β-D-1-thiogalactopyranoside [IPTG, Sangon Biotech (Shanghai, China)] was added to a final concentration of 0.2 mM. This bacterial solution was centrifuged at 37°C and 12,000 r/min for 20 min, and the cells were collected. The cells were washed in 8 mL 0.2 M phosphate buffer (pH = 7), trypsinized, and an ultrasonic disrupter (Ningkai Instrument, Nanjing, China) was used to disrupt the bacterial solution for 20 min each time, wherein each test comprises 6 times of stimulation of 4 s and rest time of 6 s. Protein expression was determined by sodium dodecyl sulfate polyacrylamide gel electrophoresis [SDS-PAGE, BIO-RAD (United States)]. Protein binding was determined by native polyacrylamide gel electrophoresis [Native-PAGE, BIO-RAD (United States)].

### 2.5 Fermentation of the recombinant strains

The verified strains were inoculated into a medium containing kanamycin (50 μg/mL) and ampicillin (100 μg/mL) in 50 mL liquid LB medium and grown at 37°C for 8–12 h. One milliliter of this culture was used to inoculate 100 mL sterilized liquid fermentation medium and grown until OD_600_ = 0.8, followed by induction at 28°C using 0.2 mM IPTG. Fermentation was carried out for 60 h, and samples were collected to measure β-alanine production by high-performance liquid chromatography (Pawellek et al., 2020); the glucose and L-lysine content was determined using a biosensor analyzer (Yanhe Biotechnology, Jinan, China). We also optimized the induction temperature. *E. coli* CGMCC 1.366-C was induced at 22°C, 25°C, 28°C, 31°C, and 34°C at 200 r/min for 20 h to explore the optimal induction temperature for the fermentation of the engineered *E. coli* strains.

Fermentation was performed in a 5 L fermenter by inoculating the activated strain in 200 mL liquid LB culture medium containing kanamycin (50 μg/mL) for 8–12 h, then 10% of the culture was used to inoculate 5 L sterilized liquid fermentation medium. It was confirmed that the previous inducer, 0.2 mM IPTG, was replaced by 5 g/L lactose. Lactose inducer was added when OD_600_ = 3 and fermented at 28°C for 20 h and then fermented at 37°C for 40 h. Samples were periodically taken to detect β-alanine.

β-alanine was detected by high performance liquid chromatography (HPLC). First, the sample was treated with 2,4-dinitrofluorobenzene (DNFB) for pre-column derivatization (Qu et al., 2021). One hundred microliters of the sample was taken, followed by the sequential addition of 100 μL 0.5 mol/L sodium bicarbonate solution (pH = 9.0) and 100 μL 1% 2,4-dinitrofluorobenzonitrile. The solution was mixed well, and placed in a water bath protected from the light at 60°C for 1 h, cooled at room temperature, followed by the addition of 700 μL 5 mM phosphate buffer (pH = 7.0), and centrifuged at 12,000 r/min for 5 min. The supernatant was passed through a 0.22 μm microporous membrane, and transferred to a liquid chromatography sample vial for later use. The HPLC instrument was high performance liquid chromatography-1 (Shimadzu LC-20 A, Japan). The chromatographic column was a Shim-pack GIST C18, and the sample was separated using an isocratic method using 1:1 mobile phase A:B, where mobile phase A and B is sodium acetate (2.5 g/L sodium acetate, 1.5 mL acetic acid, and ultrapure water to 1 L) and pure methanol, respectively. The flow rate was 1 mL/min, the column temperature was 40°C, the injection volume was 10 μL, the ultraviolet wavelength was 360 nm, and the detection time was 20 min.

## 3 Results

### 3.1 Effects of the *lysC* gene knockout in *Escherichia coli* CGMCC 1.366

CGMCC 1.366 is an *E. coli* engineered bacterium that has a yield of L-lysine, and aspartate kinase plays a decisive role in the synthetic metabolic pathway of L-lysine. Aspartate kinase was knocked out to block the synthesis of L-lysine to improve the accumulation of β-alanine. The aspartate kinase gene knockout strain (*E. coli* CGMCC 1.366-A) was obtained after homologous recombination, positive selection, and PCR detection. The upper and lower homology arms (*lysC*1 and *lysC*2) and the FRT site fragment were (FRT-Kan-FRT) amplified by PCR. The agarose gel electropherograms of the fragments are shown in Supplementary Figure S3A–C. Lanes 1–4 in Supplementary Figure S3A are agarose gel electropherograms of the PCR-amplified homologous arm fragment on *lysC*, which is 500 bp in length. Lanes 1–4 in Supplementary Figure S3B are agarose gel electropherograms of the PCR-amplified homologous arm fragment under *lysC*, which is 500 bp in length. Lanes 1–4 in Supplementary Figure S3C are agarose gel electropherograms of the PCR-amplified FRT-Kan-FRT fragment, which is 1,324 bp in length. Lanes 1–6 in Supplementary Figure S3D are agarose gel electropherograms of the double-digested plasmid by selecting restriction enzyme sites *Nde* I and *Hin*d III, which is 5,306 bp in length. Lanes 1–6 in Supplementary Figure S3E are agarose gel electropherograms of the knockout fusion fragment, which is 2,324 bp in length. Lanes 1–6 in Supplementary Figure S3F are agarose gel electropherograms for a gene fragment validation of pKD46 plasmid, which is 775 bp in length. Lanes 1–6 in Supplementary Figure S3G are agarose gel electropherograms of gene fragments on pCP20 plasmid, which is 660 bp in length. M represents the DNA marker. *E. coli* CGMCC 1.366-A was fermented for 60 h, with a β-alanine yield of 0.529 mg/L. Under the condition of 250 mg/L L-lysine initial concentration, the L-lysine production was 1,510 mg/L after 60 h fermentation. *E. coli* CGMCC 1.366 secreted 1,260 mg/L L-lysine, and the remaining L-lysine content of *E. coli* CGMCC 1.366-A strain was 30 mg/L, the result showed that *E. coli* CGMCC 1.366-A could not synthesize L-lysine. This indicates that the *lysC* gene of *E. coli* CGMCC 1.366 was successfully knocked out.

### 3.2 Expression and assembly of recombinant *aspC* and *panD*

**FIGURE 2:**
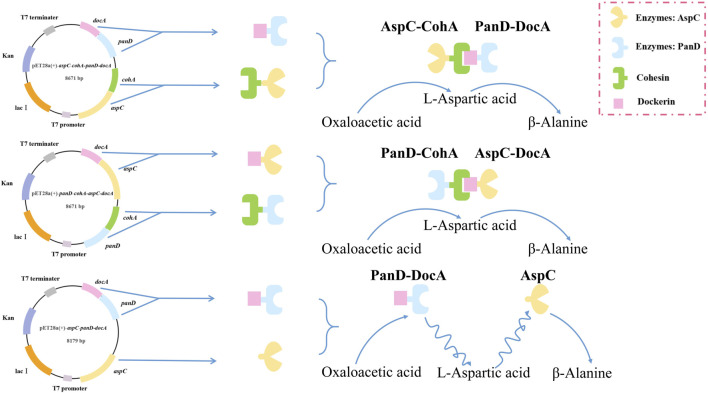
Construction of plasmid flow chart based on cellulosomes multi-enzyme self-assembly system. Three recombinant vectors are constructed, which are pET28a (+)-*aspC*-*cohA*-*panD*-*docA*, pET28a (+)-*panD*-*cohA*-*aspC*-*docA* and pET28a (+)-*aspC*-*panD*-*docA*. The key enzymes (AspC and PanD) in the metabolic pathway of the synthetic β-alanine are assembled by system of key elements of cellulosomes (cohesin and dockerin).

**FIGURE 3:**
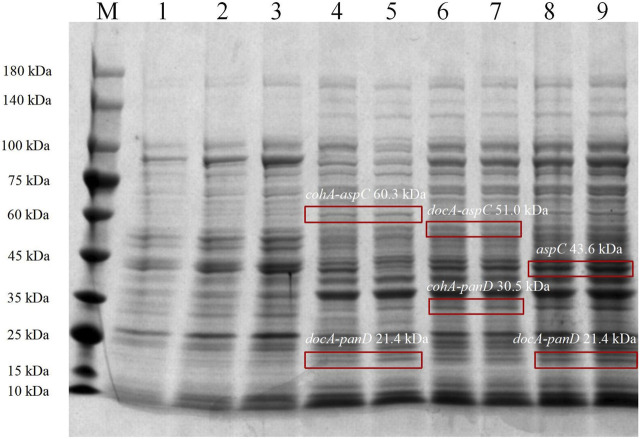
SDS-PAGE protein gel electrophoresis of *Escherichia coli* strains. SDS-PAGE protein gel electrophoresis of *Escherichia coli* CGMCC 1.366-B, CGMCC 1.366-C, and CGMCC 1.366-D were induced at 28°C for 20 h. Lanes 1–3 are protein electropherograms of control plasmid pET28a (+). Lanes 4–5 are protein electropherograms of *Escherichia coli* CGMCC 1.366-B. Lanes 6–7 are protein electropherograms of *Escherichia coli* CGMCC 1.366-C. Lanes 8–9 are protein electropherograms of *Escherichia coli* CGMCC 1.366-D.

In the metabolic pathway for β-alanine production, L-aspartate aminotransferase expression for the production of L-aspartate and co-expression with L-aspartate-α-decarboxylase for the production of the L-aspartate derivative β-alanine, the optimal enzyme for increasing β-alanine production, were selected as the L-aspartate-α-decarboxylase of *B. subtilis*. Three recombinant vectors are constructed and key enzymes in the metabolic pathway of the synthetic β-alanine are assembled by system of key elements of cellulosomes (cohesin and dockerin). This method can realize the co-expression of multiple enzymes, shorten the transfer time between enzyme molecules, improve the catalytic efficiency of the enzymes, and further increase the product yield (Figure 2). To analyze the enzymatic properties of *docA* and *cohA* binding to *panD* and *aspC* genes, the expression plasmid pET28a (+) was used. Based on SDS-PAGE gel electrophoresis data, *E. coli* CGMCC 1.366-B successfully expressed *cohA* binding *aspC* and *docA* binding *panD*. The band of the protein is clear and obvious, the molecular weight of CohA-AspC protein and DocA-PanD protein was approximately 60.3 kDa and 21.4 kDa, respectively. *E. coli* CGMCC 1.366-C successfully expressed *cohA* binding *panD* and *docA* binding *aspC*. The molecular weight of CohA-PanD protein and DocA-AspC protein was approximately 30.5 kDa and 51.0 kDa, respectively. The molecular weight of *aspC* protein and DocA-PanD protein in *E. coli* CGMCC 1.366-D was approximately 43.6 kDa and 21.4 kDa, respectively, based on SDS-PAGE protein gel electrophoresis Figure 3. The molecular weight of CohA protein and DocA protein was approximately 16.7 kDa and 6.7 kDa, respectively (Supplementary Figure S4). In Native-PAGE protein gel electrophoresis (Supplementary Figures S5, S6), the bands in the red box on lanes 3-4 are larger than the bands on lanes 1-2, indicating that AspC and PanD were assembled in *E. coli* CGMCC 1.366-B and *E. coli* CGMCC 1.366-C.

### 3.3 Construction of the recombinant strain

**FIGURE 4:**
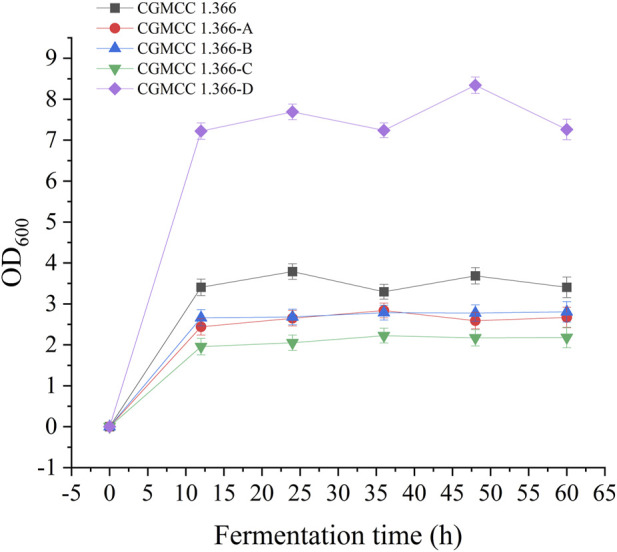
Detection of OD_600_ in fermentation of engineered *Ecoli* strains. Samples were taken from the fermentation medium every 12 h to determine the OD_600_ in CGMCC 1.366, CGMCC 1.366-A, CGMCC 1.366-B, CGMCC 1.366-C, and CGMCC 1.366-D. Standard errors are shown as bars.

**FIGURE 5:**
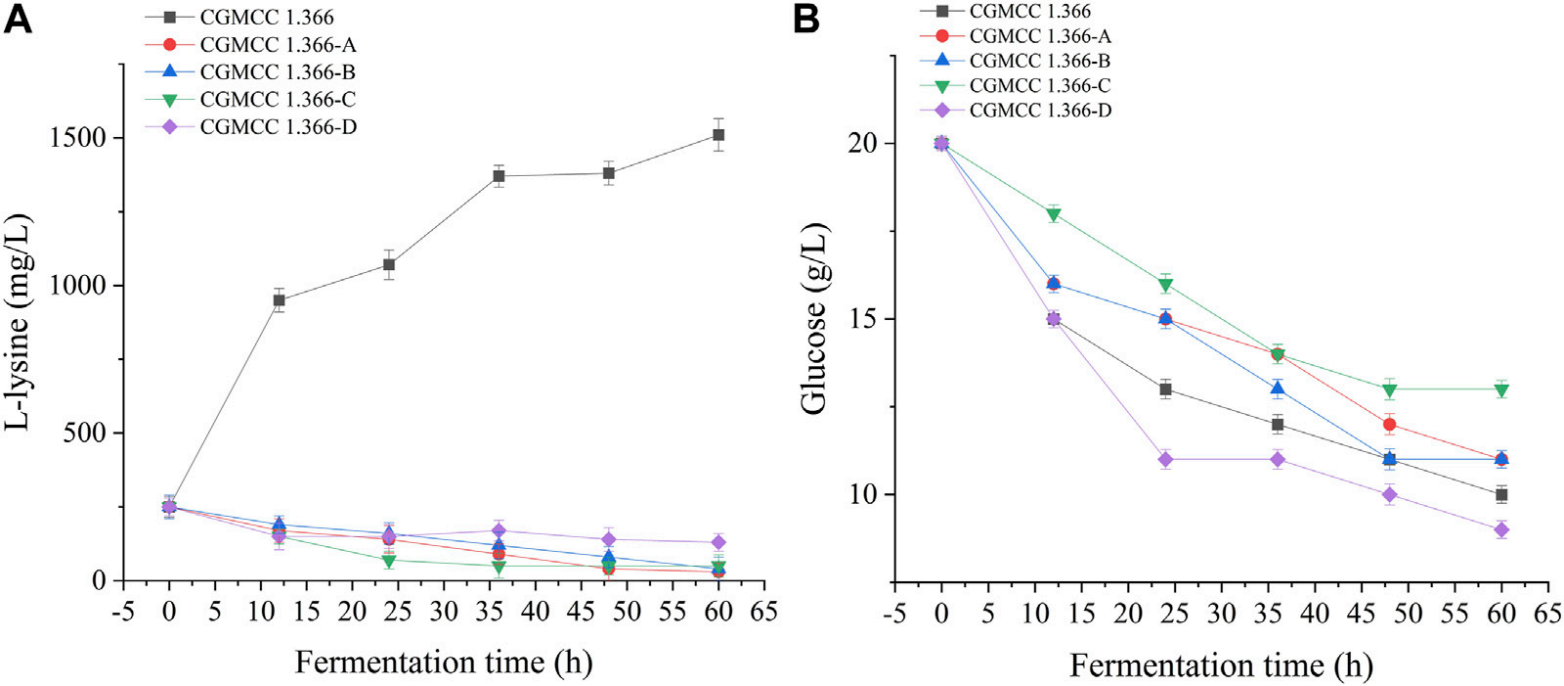
L-lysine production and glucose consumption by engineering *E. coli* strains. Samples were taken from the fermentation medium every 12 h to determine the concentration of L-lysine **(A)** and glucose **(B)** in **E. coli** CGMCC 1.366, CGMCC 1.366-A, CGMCC 1.366-B, CGMCC 1.366-C, and CGMCC 1.366-D. Standard errors are shown as bars.

**FIGURE 6:**
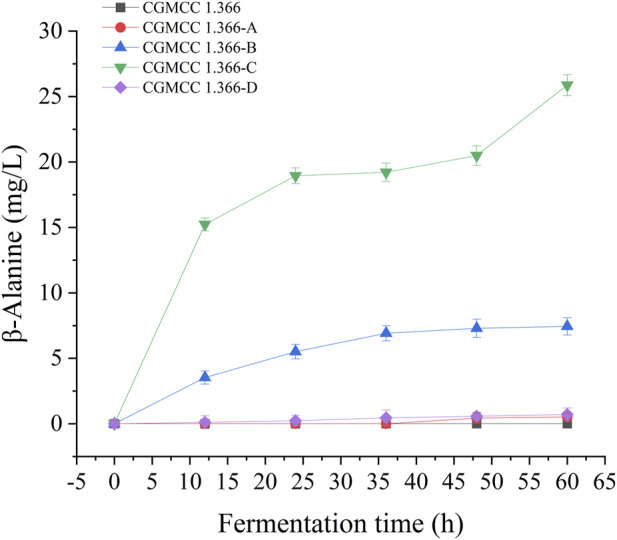
β-Alanine production by engineering *E coli* strain in fermentation medium. Samples were taken from the fermentation medium every 12 h to measure the β-alanine concentration of CGMCC 1.366, CGMCC 1.366-A, CGMCC 1.366-B, CGMCC 1.366-C, and CGMCC 1.366-D. Standard errors are shown as bars.

**FIGURE 7:**
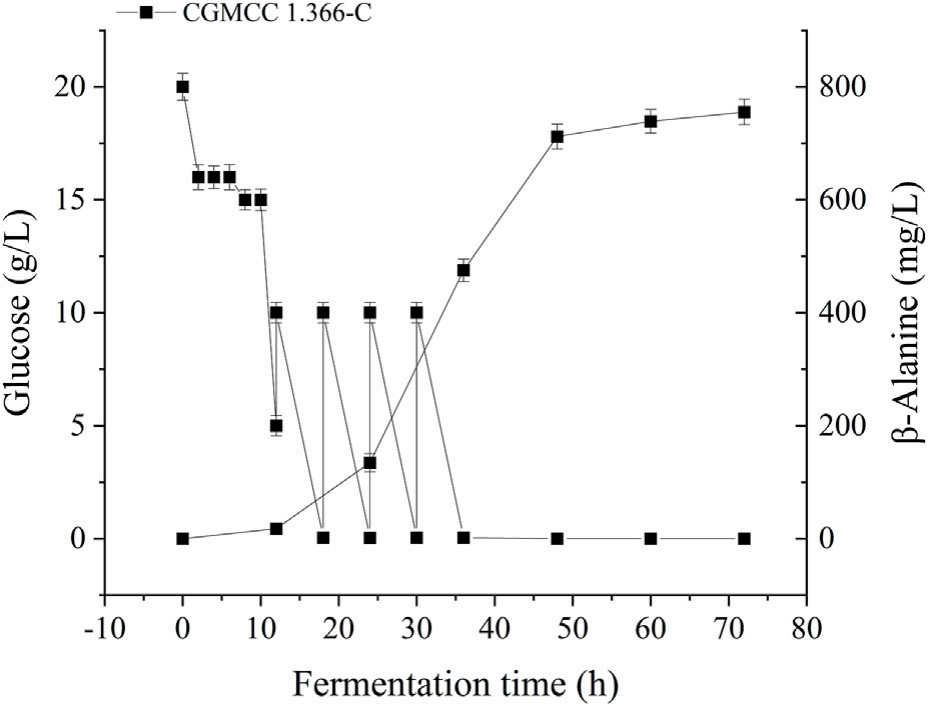
β-Alanine production and consumption of glucose by engineering *Escherichia coli* strain in a 5 L fermenter. The β-alanine and glucose concentrations in *Escherichia coli* CGMCC 1.366-D were determined. Standard errors are shown as bars.

Recombinant *E. coli* CGMCC 1.366-A was transformed with the construction plasmid pET28a (+)-*aspC-cohA-panD-docA* containing cellulosome assembly elements to construct recombinant *E. coli* CGMCC 1.366-B. The constructed plasmid pET28a (+)-*panD-cohA-aspC-docA* was transferred into recombinant *E. coli* CGMCC 1.366-A to construct recombinant *E. coli* CGMCC 1.366-C. The unassembled construction plasmid pET28a (+)-*aspC*-*panD*-*docA* was transferred into recombinant *E. coli* CGMCC 1.366-A to construct recombinant *E. coli* CGMCC 1.366-D. Recombinant *E. coli* CGMCC 1.366-B, *E. coli* CGMCC 1.366-C, and control *E. coli* CGMCC 1.366, CGMCC 1.366-A, and CGMCC 1.366-D were fermented in shake flasks. Glucose was used as the raw material in 100 mL of fermentation broth, then IPTG (0.2 mM) was added when the recombinant strain was cultured to OD_600_ = 0.8. Fermentation was carried out for 60 h, and sampling was carried out at 12 h. The OD_600_ detected by the spectrophotometer (Yuanxi Instrument, Shanghai, China) is shown in Figure 4. The residual L-lysine content and the detected glucose content obtained after the detection by the biosensor analyzer are shown in Figure 5. The content of β-alanine produced after HPLC (Shimadzu LC-20A, Japan) detection is shown in Figure 6. We found that the cell density of the five strains increased rapidly in the first 12 h. *E. coli* CGMCC 1.366-A, CGMCC 1.366-B, and CGMCC 1.366-C grew slowly compared with the wild type strain *E. coli* CGMCC 1.366 OD_600_ = 3.685. Although the unassembled strain (CGMCC 1.366-D) grew faster and consumed more glucose. Whereas the synthetic content of β-alanine was almost the same as *E. coli* CGMCC 1.366 and CGMCC 1.366-A, which could hardly produce β-alanine and the content was low, which was 0.7103 mg/L. The residual L-lysine content produced by the knockout *E. coli* CGMCC 1.366-A and the recombinant CGMCC 1.366-B and CGMCC 1.366-C was almost 0 mg/L after 60 h of fermentation at the initial concentration of 250 mg/L L-lysine, indicating that the three strains no longer synthesized L-lysine. The β-alanine yield of recombinant *E. coli* CGMCC 1.366-B and CGMCC 1.366-C was significantly improved and the β-alanine content reached 7.439 mg/L and 25.87 mg/L, respectively after 60 h compared with the control strain that hardly produces β-alanine. The β-alanine content was 0.7103 mg/L compared with control *E. coli* CGMCC 1.366-D which was not assembled. The β-alanine yield of recombinant *E. coli* CGMCC 1.366-B and CGMCC 1.366-C were 10.47 times and 36.42 times than those of the unassembled control *E. coli* CGMCC 1.366-D. In the experiment of optimizing induction temperature, *E. coli* CGMCC 1.366-C was induced by IPTG at 22°C, 25°C, 28°C, 31°C, and 34°C, respectively. The engineered *E. coli* strain produced the best β-alanine content under induction at 28°C (Supplementary Figure S7). The production performance of *E. coli* CGMCC 1.366-C with high β-alanine content in fed-batch fermentation was studied. *E. coli* CGMCC 1.366-C was induced at a final concentration of 0.2 mM IPTG and 5 g/L lactose to verify the effect of the inducer on β-alanine synthesis in a fermentation experiment. The content of β-alanine was higher under lactose induction than that under IPTG induction (Supplementary Figure S8). With the increase of fermentation time, the initial concentration of glucose of 20.0 g/L was rapidly consumed to 5 g/L after 12 h. The fermentation time after feeding was between 12 h and 36 h and feeding every 6 h, the substrate was exhausted. The results showed that the substrate consumption of *E coli* CGMCC 1.366-C was fast in 5 L fermentor. The β-alanine content was 755.465 mg/L after 72 h of fermentation (Figure 7). The HPLC chromatogram of β-alanine synthesis by fermentation of *E coli* CGMCC 1.366-C, with the peak time of 5.976 min (Supplementary Figure S9). This indicates that the assembly system of key elements of cellulosomes promotes β-alanine synthesis.

## 4 Discussion

β-alanine synthesis performed using the three-enzyme cascade method from fumaric acid utilizing L-aspartate-α-decarboxylase from *B. subtilis* resulted in a conversion rate of 90% (Wang X. et al., 2020). In this study, L-aspartate-α-decarboxylase from *B. subtilis* was used to catalyze the synthesis of β-alanine, which is catalyzed by aspartic acid. L-aspartate-α-decarboxylase and aspartate aminotransferase were combined to improve the efficiency of product synthesis by double-enzyme catalysis, and aspartate aminotransferase catalyzes the synthesis of aspartic acid by oxaloacetate. Overexpression of L-aspartate aminotransferase to produce aspartic acid and coexpression with L-aspartate-α-decarboxylase produces the L-aspartate derivative, β-alanine (Piao et al., 2019). L-aspartate and β-alanine are efficiently produced directly from glucose in *E. coli*. Fed-batch bioconversion produces 33.1 g/L L-aspartate or 37.7 g/L β-alanine. Methods to improve the catalytic efficiency of enzymes requires further study.

Cellulosome assembly is an efficient method to assemble multiple enzymes. An alcohol dehydrogenase and ω-transaminase were fused to cohesin and dockerin domains to drive their sequential and ordered coimmobilization on agarose porous microbeads. The yield of the corresponding amine was 1.3 and 10 times higher than the spatially segregated immobilized system and free enzymes, respectively using the physically colocalized enzymatic system confined to porous microbeads (Zeballos et al., 2021). In this study, a self-assembly system of key elements of cellulosomes (cohesin and dockerin) which are key element of a cellulosome combined with L-aspartate-α-decarboxylase and aspartate aminotransferase. Knockout of an aspartokinase gene in metabolic pathway of *E. coli* CGMCC 1.366 for producing L-lysine. The pathway for producing L-lysine is blocked, and the accumulation of byproducts is reduced. The key elements of the cellulosome (CohA and DocA) were successfully expressed in combination with AspC and PanD according to the Native-PAGE gel electrophoresis data. Finally, the yields of β-alanine in recombinant *E. coli* CGMCC 1.366-B and CGMCC 1.366-C were 10.47 times and 36.42 times than those of the unassembled control *E. coli* CGMCC 1.366-D. β-alanine production was increased to 755.465 mg/L following optimized fermentation compared to the yield using the original strain, which was almost 0 mg/L. The β-alanine content of *E. coli* CGMCC 1.366-C was much higher than *E. coli* CGMCC 1.366-B. *E. coli* CGMCC 1.366-C is obtained from the spatial position of AspC and PanD in *E. coli* CGMCC 1.366-B. We speculated that the amino acid sequences of the N-terminal and C-terminal changed, which affected the enzyme activity and the way the enzymes interact. The molecular weight and structure of CohA and DocA are quite different, which may affect the direction and relative position of the active site of AspC and PanD after exchange linking, thus affecting the synergistic catalysis effect of enzymes.

In conclusion, a biosynthetic pathway of β-alanine was constructed from glucose that is more suitable for industrial production and is lower in cost than the enzymatic conversion of L-aspartate or fumarate used in prior experiments. β-alanine production using double enzymes is a mature technology (Piao et al., 2019). The assembly of various β-alanine synthesis-related enzymes using cellulosome mutant elements can shorten the process of transfer and processing of intermediate metabolites between catalytic active centers of different enzymes in the microbial anabolic pathway, achieve flexible regulation of the type and expression level of enzyme proteins, and improve the catalytic efficiency of enzymes to improve the yield of the products. This is a new attempt in the microbial biosynthetic pathway. In the future, the cellulosome will be used in more various fields.

## Data Availability

The raw data supporting the conclusion of this article will be made available by the authors, without undue reservation.
