# Supplementary material for: Study on the construction technology of β-alanine synthesizing Escherichia coli based on cellulosome assembly
Source: Front Bioeng Biotechnol. 2023 Jun 2;11:1202483. doi: 10.3389/fbioe.2023.1202483 (PMC10273014; doi:10.3389/fbioe.2023.1202483)
Supplement: Supplementary file 1 [file DataSheet1.docx]

Supplementary Material

Study on the Construction Technology of β-Alanine Synthesizing *Escherichia coli* Based on Cellulosome Assembly

Jie Lu^1,2^, Guodong Wang^1,2^, Cuiping Yang^1,2^, Zehao Peng^1,2^, Lu Yang^1,2^, Bowen Du^1,2^, Chuanzhuang Guo^3^, Songsen Sui^3^, Jianbin Wang^3^, Junlin Li^3^, Ruiming Wang^1,2^, Junqing Wang^1,2*^

^1^State Key Laboratory of Biobased Material and Green Papermaking (LBMP), Qilu University of Technology, Jinan 250353, Shandong, People's Republic of China

^2^Department of Biological Engineering, Qilu University of Technology, 250353, Shandong, People's Republic of China

^3^Zhucheng Dongxiao Biotechnology Co., Ltd, Xinxing Town, Zhucheng 262200, Shandong, People's Republic of China

*** Correspondence:**Junqing Wang
wjqtt.6082@163.com

**Supplementary Table. 1** Primers used in this study

| **Primers** | **Description** | **Source** |
| --- | --- | --- |
| *lysC*1-F/R | Amplification of the upper homologous arm of the *lysC* gene | Sangon |
| *lysC*2-F/R | Amplification of the lower homologous arm of the *lysC* gene | Sangon |
| FRT-Kan-FRT-F/R | Amplification of the FRT-Kan-FRT sequence on plasmid pKD13 | This study |
| YZpkd46-F/R | Amplification of bet gene fragment from pKD46 plasmid | Sangon |
| YZcohA-F/R | PCR amplification verification | Sangon |
| YZdohA-F/R | PCR amplification verification | Sangon |

**Supplementary Figure 1**

**
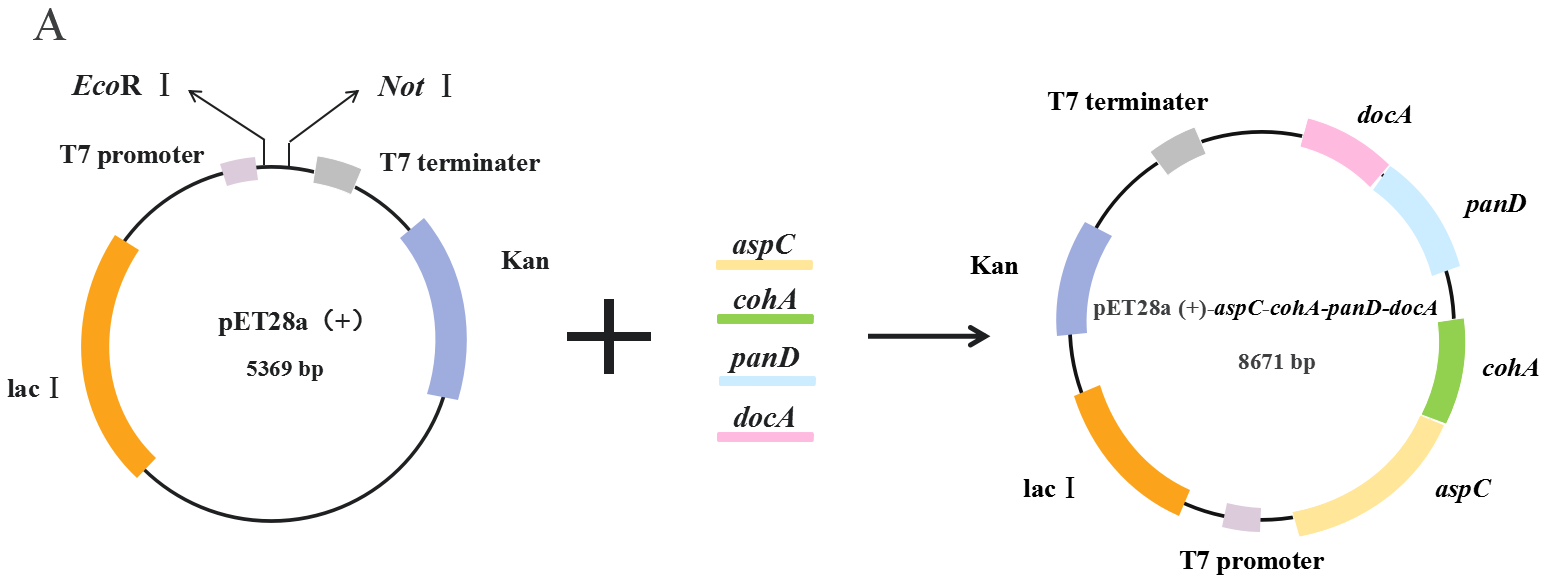
**

**
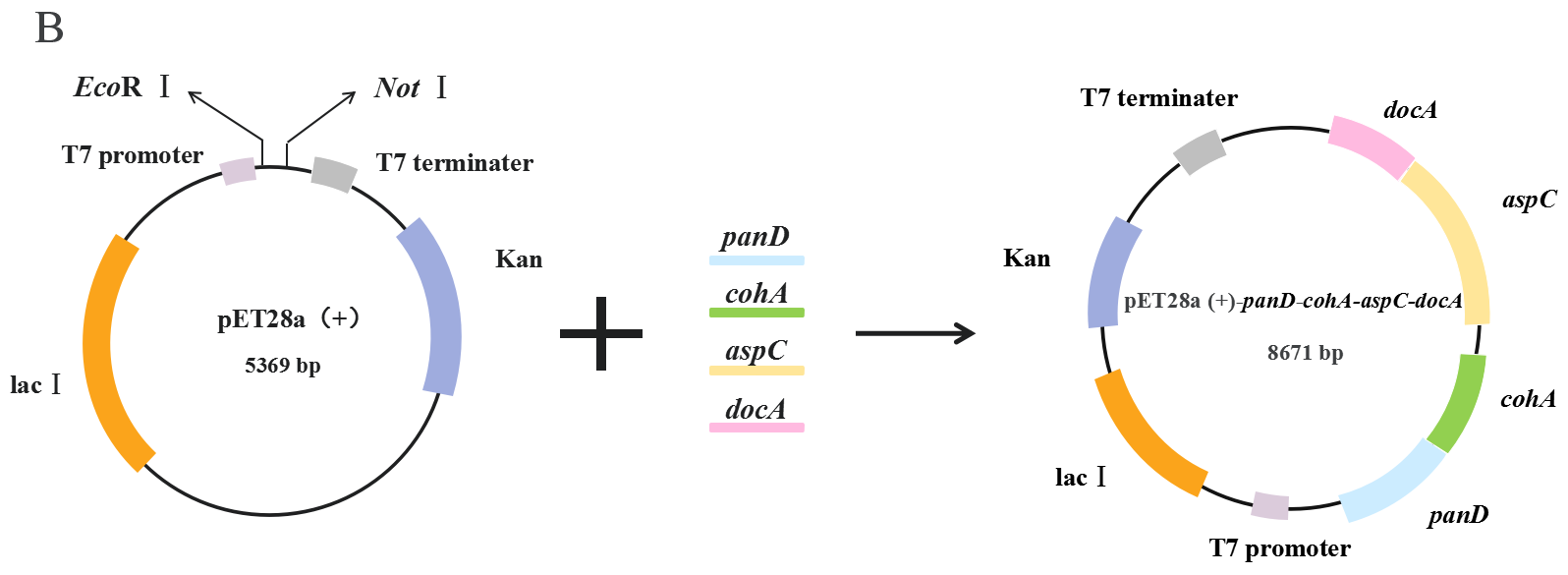
**

**
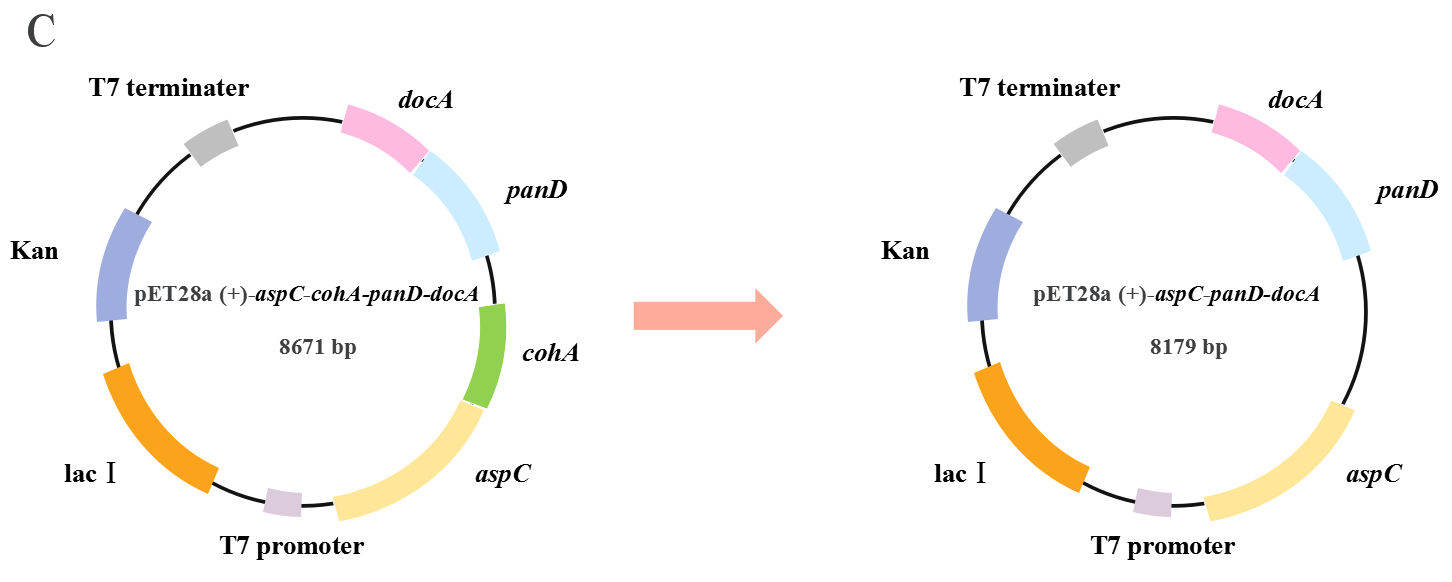
**

**Plasmid construction diagrams.** Supplementary Figure 1A shows the constructed plasmid pET28a (+)-*aspC*-*cohA*-*panD*-*docA.* Supplementary Figure 1B shows the constructed plasmid pET28a (+)-*panD*-*cohA*-*aspC*-*docA*. Supplementary Figure 1C shows the constructed plasmid pET28a (+)-*aspC*-*panD*-*docA*

**Supplementary Figure 2**

**
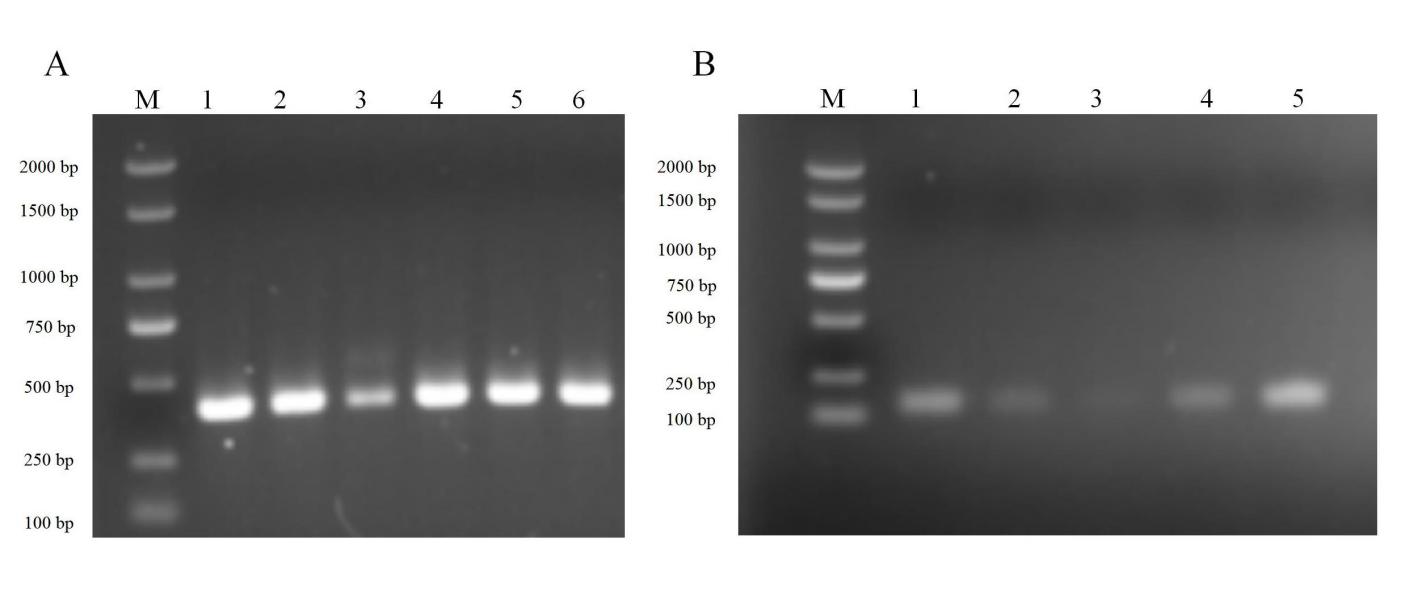
**

**The agarose gel electrophoresis of the plasmid transferred into the *E. coli* engineering bacteria was verified.** M in the figure represents Marker. In supplementary figure2A, lanes 1–3 are agarose gel electrophoresis diagrams for verifying the transformation of plasmid pET28a (+)-*aspC-cohA-panD-docA* into *E. coli* CGMCC 1.366-A and lanes 4–6 are agarose gel electrophoresis diagrams for verifying the transformation of plasmid pET28a (+)-*panD-cohA-aspC-docA* into *E. coli* CGMCC 1.366-A, which is 421 bp in length. In supplementary figure2B, lanes 1–5 are agarose gel electrophoresis diagrams for verifying the transformation of plasmid pET28a (+)-*panD-aspC-docA* into *E. coli* CGMCC 1.366-A, which is 148 bp in length.

**Supplementary Figure 3**


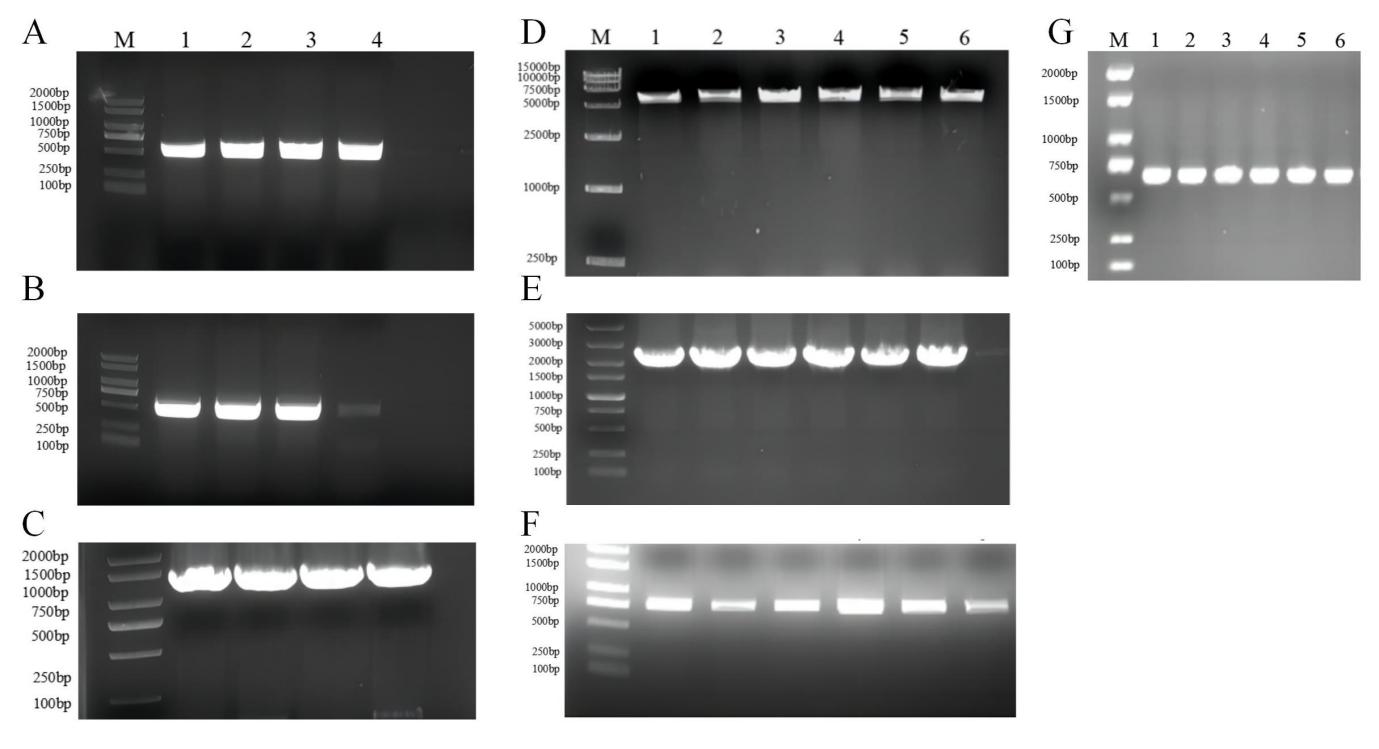


**Agarose gel electrophoresis of gene fragments for constructing knockout strains.** Lanes 1–4 in Supplementary Figure 3A are agarose gel electropherograms of the PCR-amplified homologous arm fragment on *lysC*, which is 500 bp in length. Lanes 1–4 in Supplementary Figure 3B are agarose gel electropherograms of the PCR-amplified homologous arm fragment under *lysC*, which is 500 bp in length. Lanes 1–4 in Supplementary Figure 3C are agarose gel electropherograms of the PCR-amplified FRT-Kan-FRT fragment, which is 1324 bp in length. **D, E** Agarose gel electrophoresis of double-digested plasmid by selecting restriction enzyme sites *Nde* I and *Hin*d III and knockout fusion fragment. Lanes 1–6 in Supplementary Figure 3D are agarose gel electropherograms of the double-digested plasmid, which is 5306 bp in length. Lanes 1–6 in Supplementary Figure 3E are agarose gel electropherograms of the knockout fusion fragment, which is 2324 bp in length. **F** Agarose gel electrophoresis for bet gene validation. Lanes 1–6 in Supplementary Figure 3F are agarose gel electropherograms for a gene fragment validation of pKD46 plasmid, which is 775 bp in length. **G** Agarose gel electrophoresis of gene fragments in the pCP20 plasmid. Lanes 1–6 in Supplementary Figure 3G are agarose gel electropherograms of gene fragments on pCP20 plasmid, which is 660 bp in length. M represents the DNA marker.

**Supplementary Figure 4**

**
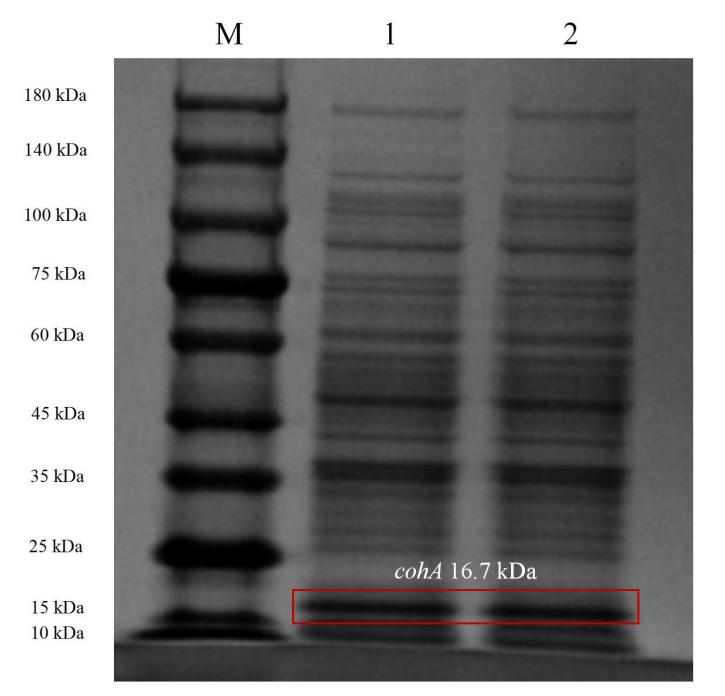
**

**SDS-PAGE protein gel electrophoresis of *E. coli* strains.**

The band marked with red box is CohA 16.7 kDa. The molecular weight of DocA protein is too small, only 6.7 kDa, to be displayed in the protein band.

**Supplementary Figure 5**

**
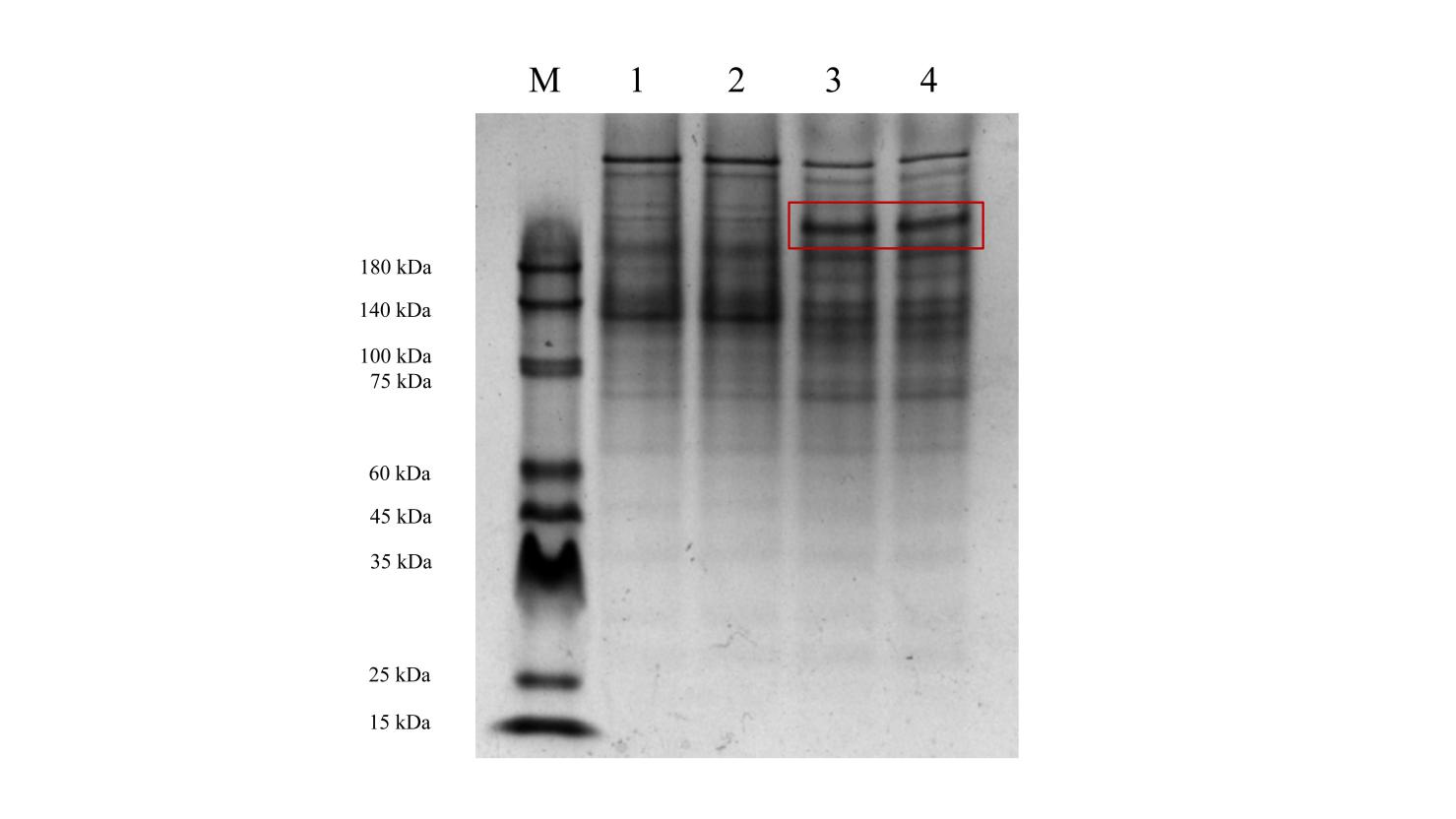
**

**Native-PAGE protein gel electrophoresis of *E. coli* strains.**

The first and second lanes in the figure represent protein bands induced by *E. coli* CGMCC 1.366-D, which the AspC and CohA were not assembled. The third and fourth lanes represent protein bands induced by *E. coli* CGMCC 1.366-B, which assembles by CohA and DocA. The bands in the red box on lanes 3-4 are larger than the bands on lanes 1-2.

**Supplementary Figure 6**

**
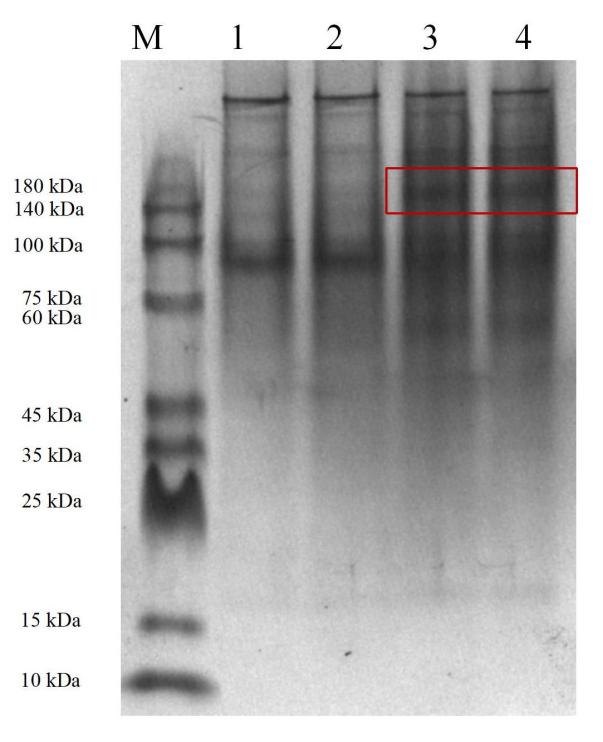
**

**Native-PAGE protein gel electrophoresis of *E. coli* strains.**

The first and second lanes in the figure represent protein bands induced by *E. coli* CGMCC 1.366-D, which the AspC and CohA were not assembled. The third and fourth lanes represent protein bands induced by *E. coli* CGMCC 1.366-C, which assembles by CohA and DocA. The bands in the red box on lanes 3-4 are larger than the bands on lanes 1-2.

**Supplementary Figure 7**

**
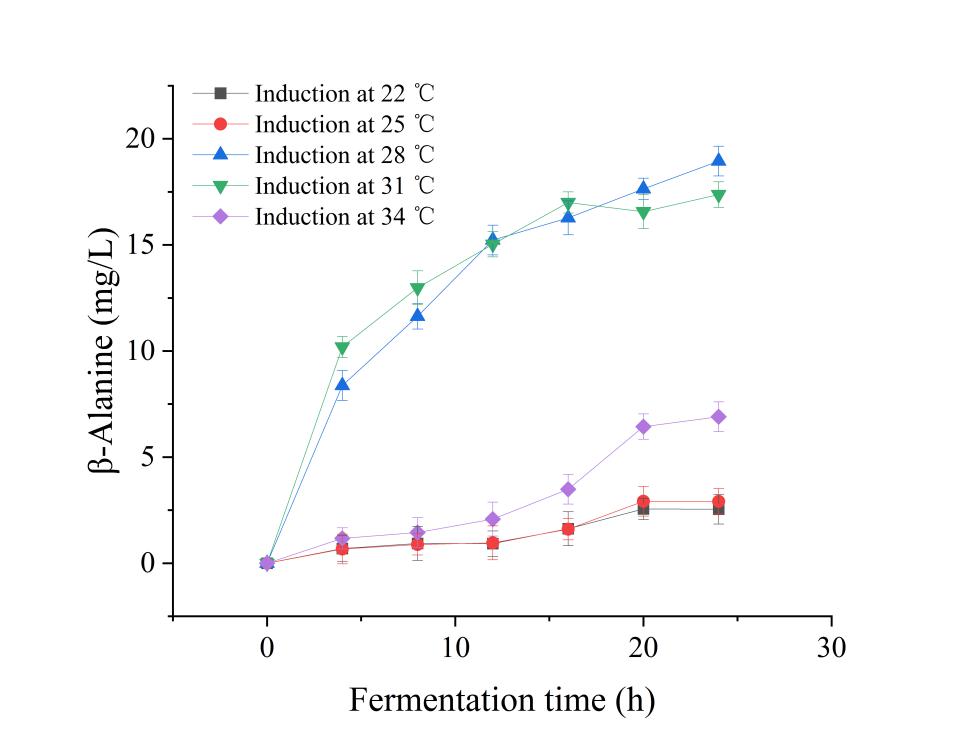
**

**The content of β-alanine induced by *E. coli* CGMCC 1.366-C at 22 ℃, 25 ℃, 28 ℃, 31 ℃ and 34 ℃, respectively.**

**Supplementary Figure 8**

**
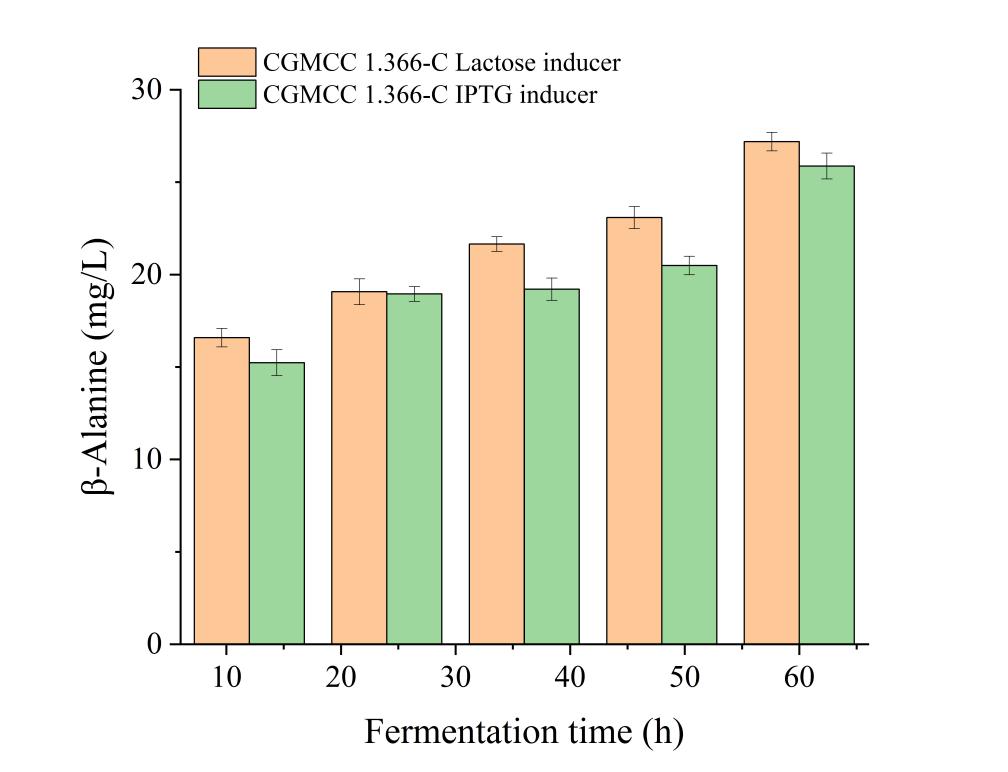
**

**Effect of inducers on β-alanine production by engineered *Ecoli* CGMCC 1.366-C.**

**Supplementary Figure 9**

**
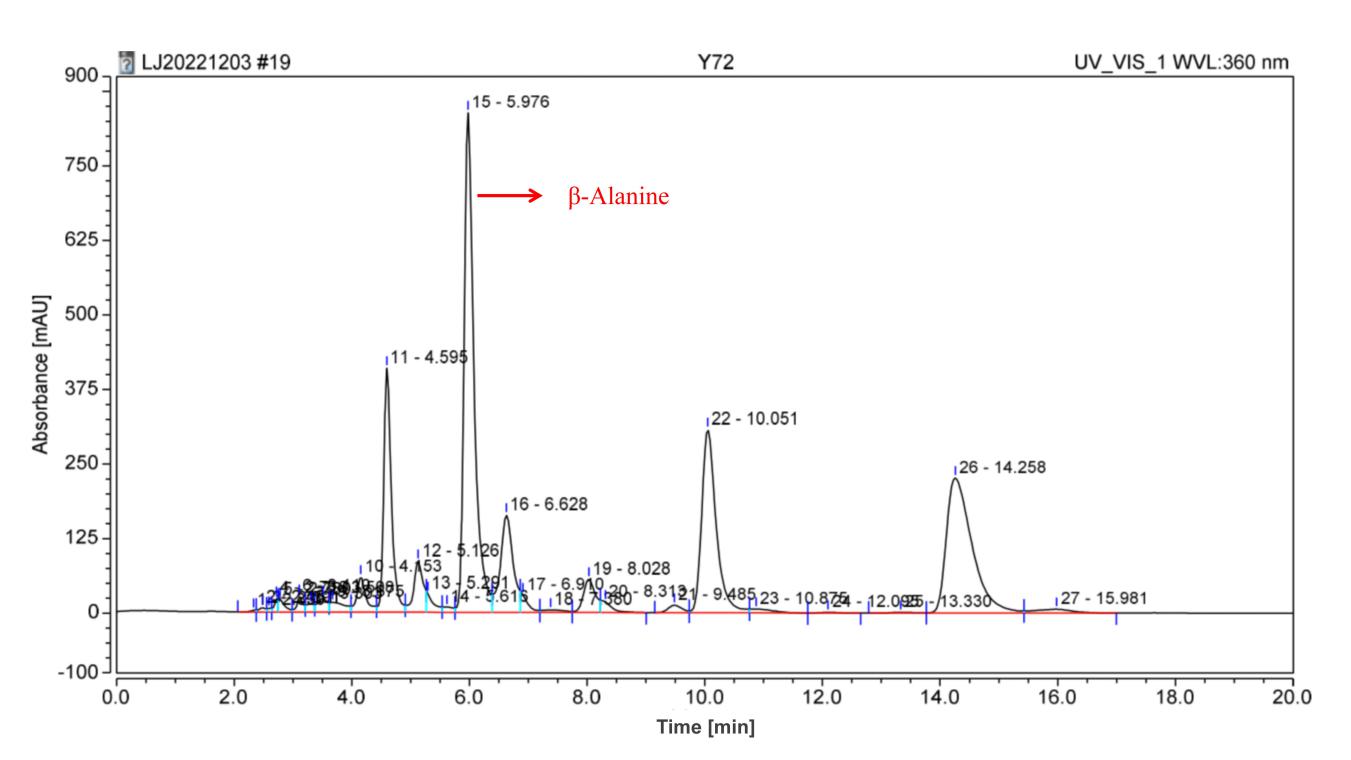
**

**High performance liquid chromatogram of β-alanine synthesized by engineered *E.coli* CGMCC 1.366-C.**
